# A dog oviduct-on-a-chip model of serous tubal intraepithelial carcinoma

**DOI:** 10.1038/s41598-020-58507-4

**Published:** 2020-01-31

**Authors:** Marcia de Almeida Monteiro Melo Ferraz, Jennifer Beth Nagashima, Bastien Venzac, Séverine Le Gac, Nucharin Songsasen

**Affiliations:** 1 Center for Species Survival, Smithsonian National Zoo and Conservation Biology Institute, 1500 Remount Road, Front Royal, Virginia 22630 USA; 2 Applied Microfluidics for Bioengineering Research, MESA+ Institute for Nanotechnology and TechMed Center, University of Twente, 7500 AE Enschede, The Netherlands

**Keywords:** Tissue engineering, Ovarian cancer, Cancer models, Genetic engineering, Lab-on-a-chip

## Abstract

Ovarian cancer is the fifth cause of cancer-related mortality in women, with an expected 5-year survival rate of only 47%. High-grade serous carcinoma (HGSC), an epithelial cancer phenotype, is the most common malignant ovarian cancer. It is known that the precursors of HGSC originate from secretory epithelial cells within the Fallopian tube, which first develops as serous tubal intraepithelial carcinoma (STIC). Here, we used gene editing by CRISPR-Cas9 to knock out the oncogene *p53* in dog oviductal epithelia cultured in a dynamic microfluidic chip to create an *in vitro* model that recapitulated human STIC. Similar to human STIC, the gene-edited oviduct-on-a-chip, exhibited loss of cell polarization and had reduced ciliation, increased cell atypia and proliferation, with multilayered epithelium, increased *Ki67*, *PAX8* and *Myc* and decreased *PTEN* and *RB1* mRNA expression. This study provides a biomimetic *in vitro* model to study STIC progression and to identify potential biomarkers for early detection of HGSC.

## Introduction

Ovarian high-grade serous carcinoma (HGSC) and tubal epithelium share similar morphology, immuno-phenotype, and gene and protein expression patterns^1–3^. Transformed human Fallopian tube stem cells give rise to tumors that recapitulate the morphology and gene expression of HGSC after injection into immune-deficient mice^4^. Furthermore, most Fallopian tube cancer lesions (96%), such as serous tubal intraepithelial carcinoma (STIC), are marked by mutant *p53*, as are their metastatic form HGSC^5,6^. Past research on HGSC has used human tumor cells, but it has been hampered by the inefficiency of platting and sub-culturing of Fallopian tube epithelium, a process that involves a long period of fibroblast contamination reduction^7^. Furthermore, these processes result in the selection of specific cell populations, which lack tumor molecular characteristics, mutations and intra-patient heterogeneity^7–9^. Human HGSC has also been studied using patient-derived xenografts, but these technologies are time and resource-consuming, are poorly suitable for genetic manipulation and drug screening, and experience rapid mouse-specific tumor evolution^7^.

Alternatively, knocked-out animals are used, being rodents the most used genetic engineered models of HGSC^10–14^. However, data generated from rodents are not readily translated to the human. First, the mouse does not naturally develop HGSC^15,16^. Secondly, genetically engineered and syngeneic mouse models may not exhibit genetic alterations relevant to human HGSC and, in many instances, may be driven by inappropriate regulatory sequences and promoters^15,16^. Third, cancer phenotypes can vary depending on the mouse strain used to create the cancer model^16^. Fourth, infertility resulting from ovarian tumors has limited the production of transgenic mouse models for ovarian cancer^17^. Nevertheless, the recent advances of the CRISPR-Cas9 system for knocking in and out target genes might offer the same opportunity of gene editing large mammalian species and can specifically increase the efficiency of genetically engineered *in vitro* models.

Although different genetically engineered mouse models can sufficiently recapitulate HGSC, as extensively reviewed in^8,15,16,18^, and are useful for studying mechanisms of disease *in vivo*, alternative large mammalian reliable *in vitro* models are still necessary for ethical (reducing the use of animal in experimentation) and financial (having more biomimetic *in vitro* models without the need of maintaining animal colonies) reasons. The domestic dog (*Canis familiaris*) is an excellent model for human diseases, including cancer^19,20^. The histological and clinical presentation of numerous canine cancers closely parallels that of corresponding cancers in humans^21^. Furthermore, canine cancers share evolutionarily conserved genomic changes that are found in humans^21^. Interestingly, several of the human cancer predisposition genes have been discovered in the constitutional (germline) DNA of dogs with cancer, including mutations in *BRCA1*, *BRCA2*, and *p53*^21–24^. Like its human counterpart, canine *p53* is also found to be mutated in several types of dogs tumors (including osteosarcoma and mammary tumors)^25^. The dog also spontaneously develops ovarian cancer^26^ and the incidence of ovarian tumors in dogs varies from 6 to 11%, epithelial tumors being dominant (50–60%)^26–29^. This frequency is still likely underestimated, because (1) most dog ovarian epithelial tumors fail to express detectable symptoms^27,30^ and (2) family dogs are commonly ovariohysterectomized (spayed) early in life in the United States. Lastly, oviductal tissue for research can easily be collected after routine ovary-hysterectomy. This strategy provides substantial amounts of surplus tissue from a highly heterogeneous population, thereby mimicking a random human cohort, which also minimizes the use of live animals and avoids the need to manage expensive animal colonies. Similarly to other mammals, the canine oviduct is composed of a mucosal layer displaying varying heights of folding^31^. This layer is lined by a simple cuboid-to-columnar epithelium exhibiting two major cell types: ciliated and secretory cells^31^. The lamina propria is made of a cell-rich connective tissue and is followed by a sheet of several layers of smooth muscle cells, which is decreased in thickness and compactness in the infundibulum area^31^. Mast cells, lymphocytes and neutrophils can be detected within the connective tissue during all stages of reproductive cycle^31^. The numbers of ciliated and secretory cells vary among oviductal region and estrous stage^31^. The lowest number of ciliated cells is seen during anestrus in the isthmus area (<1%) and the highest in the infundibulum during late follicular and mid-luteal phase (>60%)^31^.

Altogether current *in vivo* rodent models and 2D *in vitro* models exhibit essential limitations to study human HGSC/STIC, for which the dog is a relevant model and discarded dog oviduct tissues are abundantly available. In that context, an organ-on-a-chip approach for a dog oviduct is expected to fulfill the gap between current models and the human *in vivo* situation. Organ-on-a-chip platforms are microscale advanced *in vitro* models that have brought novel capabilities to engineer levels of cell organization, differentiation and interaction that cannot be readily achieved by conventional static 2D cultures^32^. Specifically, this organ-on-a-chip technology has proven to be suitable to create sophisticated *in vitro* models of various organs to study mammalian organ-specific physiology, and/or examine different aspects of disease and toxicology^32–35^. Of particular interest here, an oviduct-on-a-chip that mimics the *in vivo* oviductal epithelial cells and is responsive to hormonal changes similar to those observed during the estrus cycle, has been described for the cow^36,37^. Additionally, a female reproductive tract-on-a-chip, which included ovarian, Fallopian tube, endometrial, cervix and hepatic cultures (EVATAR) was used to mimic the 28-day woman’s menstrual cycle^38^. However, gene editing of oviductal cells is limited in platforms like the EVATAR, which employ explants instead of cells layers^38^.

Typically, the fabrication of these complex organ-on-a-chip systems necessitates access to specialized clean-room facilities or microfluidics laboratories^39^. Critically, compartmentalized organs-on-a-chip such as the EVATAR^38^, and models for the oviduct^36,37^, lung and liver^34^, are fabricated by assembling the microfluidic devices including an intermediate thermoplastic track-etched membrane (*e.g*. polycarbonate (PC) or polyethylene terephthalate (PET)), which also requires dedicated equipment. Therefore, to utilize these promising organs-on-a-chip to create disease models, broadly accessible and rapidly prototyping methodologies must be developed.

To expand the use of promising gene-edited organ-on-a-chip disease models, we introduce a process for fabricating complex compartmentalized microfluidics devices using equipment which is ubiquitous in biological laboratories (scale, oven, and desiccator). The resulting oviduct-on-a-chip devices were used to create a biomimetic oviduct-on-a-chip, using the dog as a model. Finally, the knock out of the *p53* gene in our dog oviduct-on-a-chip model by CRISPR-Cas9 resulted in an *in vitro* platform that recapitulated the human serous tubal intraepithelial carcinoma (STIC).

## Results

### Oviduct-on-a-chip design, fabrication and epithelial cells culture

**Figure 1:**
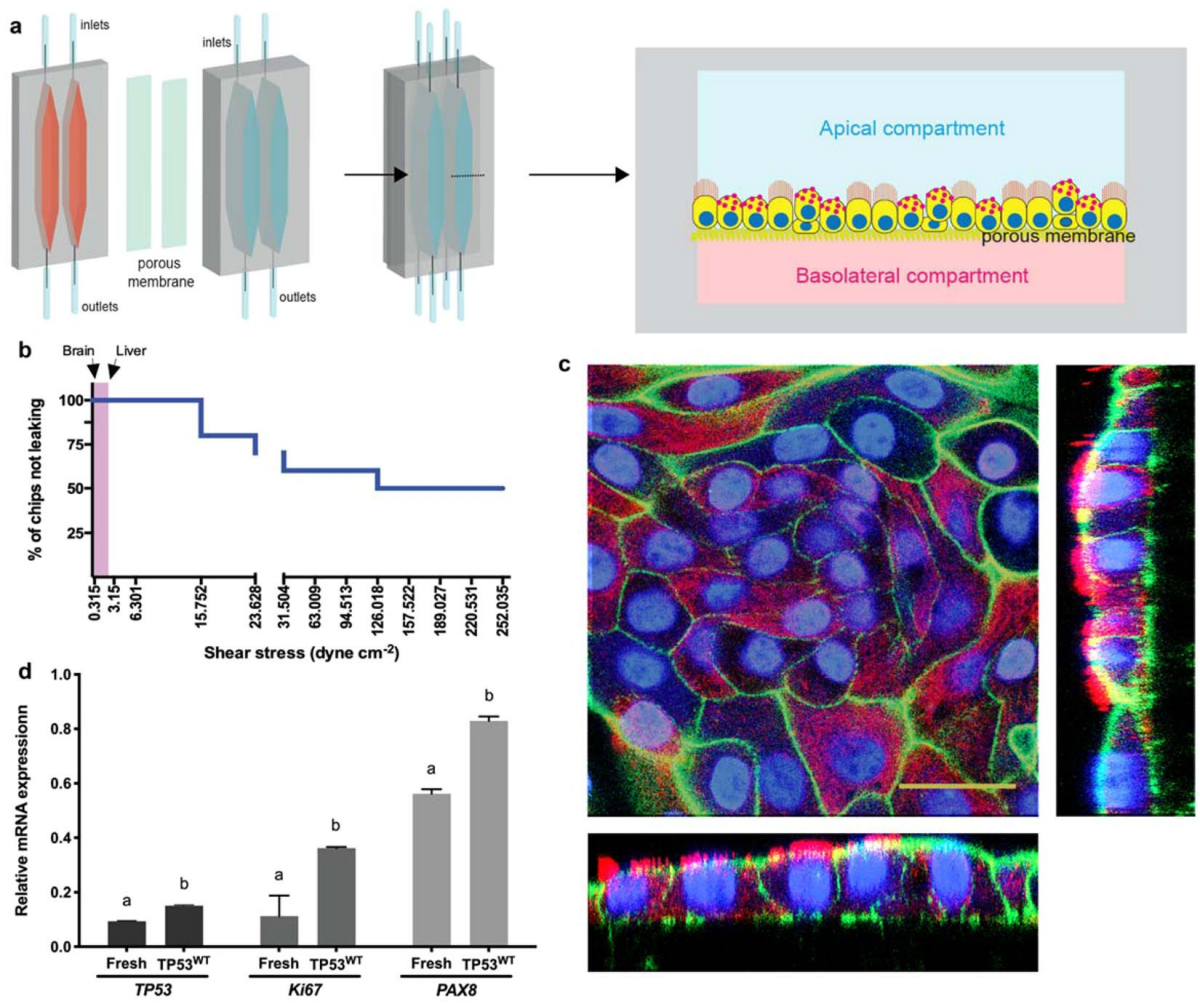
Oviduct-on-a-chip model, leakage test and epithelial cell culture. In (**a**) different parts of the device with bottom (basolateral, pink) and top (apical, blue) compartments, and the assembled microfluidic device with a porous membrane in the middle; each device comprises two independent chambers. Right graphic shows a transverse cut of the chamber, indicating the oviductal epithelium growing on the top of the porous membrane in the apical compartment. In (**b**) leakage test showing the percentage of devices not leaking as a function of the applied shear stress; note lower shear stress used in brain- and liver-on-a-chip platforms^41,42^. In (**c**), immunofluorescence with reconstructed side-views of TP53^WT^cells grown in the oviduct-on-a-chip for 2 weeks showing nuclei (Hoechst33342, blue), actin filaments (phalloidin, green), and cilia (acetylated alpha tubulin, red). In (**d**), relative mRNA expression of *TP53, Ki67* and *PAX8* of fresh collected cells and non-edited cells cultured in the oviduct-on-a-chip for 2 weeks. Scale bar = 25 µm.

For creating the dog oviductal epithelium culture, we adapted our previous bovine oviduct-on-a-chip model^36,37^. The device, made in PDMS (polydimethylsiloxane), an elastomeric silicone material, comprised a thermoplastic porous membrane on which cells were seeded. The membrane separated a basolateral chamber and a higher apical chamber (300 µm) allowing the establishment of an air-liquid interface after gene editing of the epithelium, with no fluid on the top of the epithelium, but with a flow in the basolateral compartment, mimicking the blood circulation (Fig. 1a). However, our previous fabrication method required access to cleanroom facilities to produce the devices, which is an essential limitation for the massive adoption of these organ-on-a-chip devices. Therefore, here we introduce a novel fabrication methodology relying on partial curing of PDMS parts from 3D printed molds, followed by bonding to the intermediate thermoplastic membrane, using only an oven and a desiccator. While such a partial curing approach has been already used to assemble several PDMS substrates^40^, to the best of our knowledge, it has never been attempted for bonding PDMS and thermoplastic membranes. As a first control, the bonding strength of these devices was evaluated using a leaking test, revealing the devices can resist a maximum shear stress of 252.035 dyne.cm^−2^, which corresponds to a flow rate of 8 ml min^−1^ (Fig. 1b). Importantly, these values are not very different from those obtained for devices assembled with plasma activation and silanization, demonstrating that the herein reported methodology is well-suited for organ-on-chip applications that typically utilize lower shear stress and pressure rates^34,37,41,42^.

We next cultured non-edited dog oviduct epithelial in our oviduct-on-a-chip for 3 weeks; cells exhibited similar morphology to the *in vivo* oviduct, which is characterized by a simple cuboidal to columnar epithelium containing both ciliated and secretory cells^43^, and an average cell height of 13.5 ± 3.7 and 12 ± 4.1 µm at weeks 2 and 3, respectively (Fig. 1c). Epithelial cells cultured in the oviduct-on-a-chip for 2 weeks showed increased p53, Ki67 and PAX8 mRNA expression, when compared to freshly collected cells from the same animals (Fig. 1d). As also evidenced by the higher PAX8 expression, secretory cells were the most abundant cells (90 ± 4% and at 87 ± 6% for weeks 2 and 3, respectively). Similarly, such an air-liquid organ-on-a-chip model with basolateral perfusion was already described to promote epithelial cell polarization and differentiation of human alveolar epithelium^34^.

### CRISPR-Cas9 effectively promotes editing of the p53 gene in the oviduct-on-a-chip

**Figure 2:**
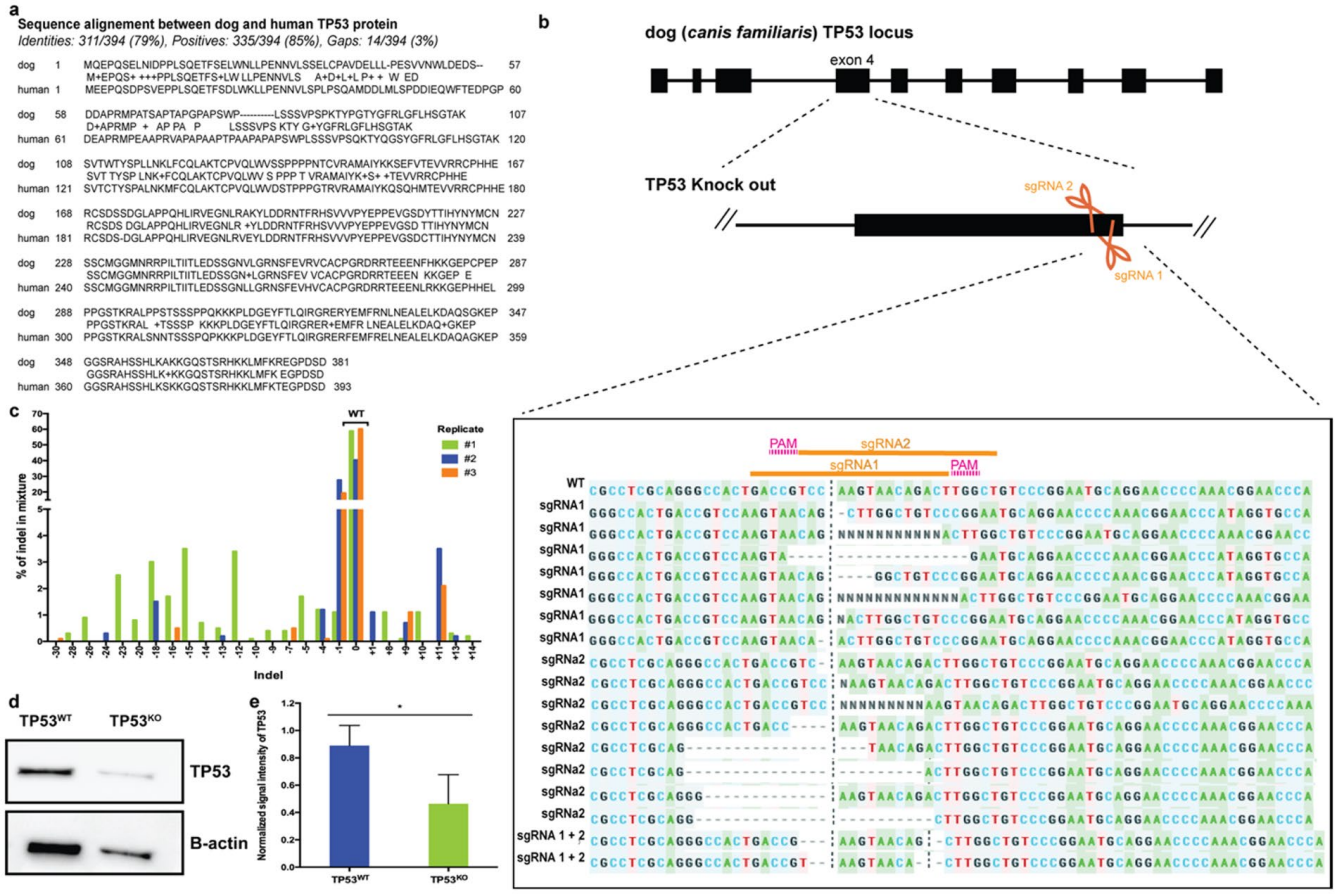
Genetic manipulation of the oviductal cells. In (**a**), comparison of human and dog TP53 proteins, showing 79% homology in their sequence. (**b**), CRISPR-Cas9 mediated editing of TP53 gene in the oviduct-on-a-chip with a representation of all Insertions and Deletions (Indels) encountered by editing with both sgRNA 1 and 2 simultaneously. (**c**) depiction of the different Indels percentage. (**d**) section of Western blot for the TP53 gene in the WT and KO cells (full gel image and negative control presented as Supplementary Fig. S1). And (**e**) TP53 protein content normalized to B-actin (n = 4, paired sample t-tests, p = 0.024).

To edit the canine *TP53* gene using the CRISPR-Cas9 system, two sgRNAs were selected based on their lowest off-target potentials as determined by two criteria: (1) sgRNAs with less binding potential (<30) to the canine genome - transcripts were sorted according to the Basic Local Alignment Search Tool (BLAST) and potential off-target genes can be seen in Supplementary Table S1; and (2) sgRNAs with high on-target score (>0.5) and at least 3 mismatched bases with off-target genes (Supplementary Tables S2 and S3), as determined by Synthego’s bioinformatics CRISPR design tool. Different insertions and deletions (Indels) originating after cleavage by sgRNA 1 and/or 2 were observed using the Inference of CRISPR Edits tool (ICE, Synthego, Fig. 2b,c)^44^. Indels varied from up to 30 base deletions and 14 base insertions, being the one base deletion the most observed Indel. The editing efficiency varied from 40 to 59%, with an average of 47% (n = 4 pools, 2 devices/pool). Our results are in good agreement with a human iPSCs study that used the same method of transfection^45^. Remarkably, the editing efficiency obtained in the present study was nearly two times higher than when human Fallopian tube cells were transfected by electroporation and using plasmid deliver of Cas9 and gRNA (47 *vs*. 25%)^7^.

As a result of the 47% editing efficiency, a similar decrease (48%) in the detection of the p53 protein level (which shares 79% sequence homology with the human p53^25^; Fig. 2a) was observed in the edited cells (TP53^KO^) when compared to the control cells (TP53^WT^) (Fig. 2d,e).

### TP53^KO^ cells lose normal morphology and exhibit increased proliferation and DNA double strand breaks

**Figure 3:**
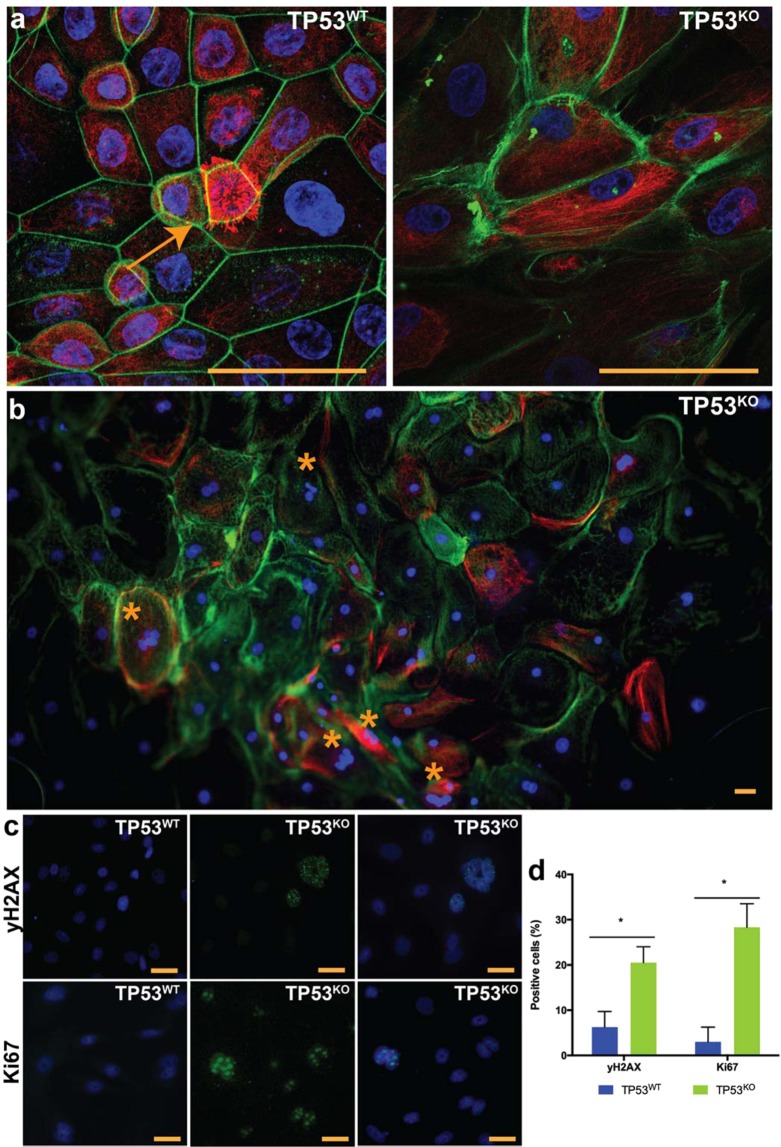
Characterization of the gene-edited oviductal cells. In (**a**,**b**) cell morphology of control (TP53^WT^) and edited cells (TP53^KO^) showing actin filaments (Phalloidin, green) nuclei (Hoechstt33342, blue) and tubulin (Acetylated tubulin, red); note the presence of ciliated cell (arrow) in TP53^WT^; and the presence of giant multi-nucleated cells (*) in TP53^KO^. In (**c**) immunofluorescence for double-strand breaks (yH2AX, green) and cell proliferation (Ki67, green) and nuclei (Hoechst33342, blue); and the percentage of positive cells for those markers (**d**). * indicates that differences are statistically significant (paired sample t-test, p < 0.05). Scale bars = 10 µm.

When mimicking STIC by editing the *p53* gene in the oviduct-on-a-chip (TP53^KO^), it was observed that cells became larger and flattened, indicating loss of cell polarity (Fig. 3a)^46^. A reduction in the number of ciliated cells was also observed, and could be explained by increased proliferation of non-ciliated cells, which is evidenced by higher *Ki67* and *PAX8* mRNA expression in the TP53^KO^ cells. Differently, the control oviduct-on-a-chip (TP53^WT^) had similar morphology to the *in vivo* oviduct (Fig. 3a). In addition to the loss of polarity, STIC is also characterized by increased cell atypia, cell proliferation, and DNA damage^2,13,46–51^. In the present study, the dog TP53^KO^ oviduct-on-a-chip had a significant increase in the number of giant multinucleated cells (9.69% *vs*. 1.24%; paired sample t-test, p = 0.020; Fig. 3b); cell proliferation, as seen by a dramatic increase in the *Ki67* expression from 2.99 ± 3.27 to 28.33 ± 5.19% (paired sample t-test, p = 0.017; Fig. 3c,d); and a higher number of cells with double-strand breaks (20.52 ± 3.51 *vs*. 6.25 ± 3.46, paired sample t-test, p = 0.007; yH2AX staining, Fig. 3c,d). The gene-edited cells also presented areas of multilayer cell growth, which is commonly seen in STIC pathologies^48–50^ but which were not observed in non-edited cells (Supplementary Fig. S2).

### TP53^KO^ express specific mRNAs reminiscent of HGSC

**Figure 4:**
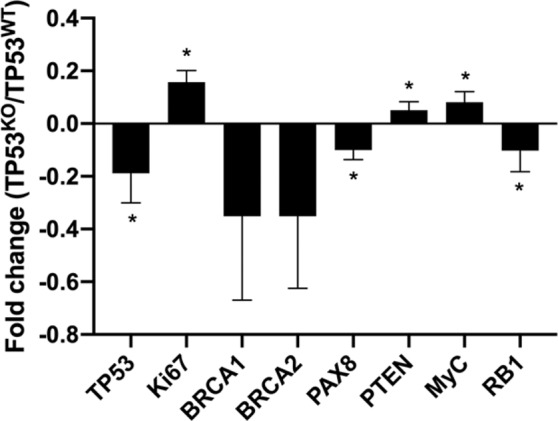
Fold change of relative mRNA expression of *TP53*, *Ki67*, *BRCA1/2*, *PAX8*, *PTEN*, *My*c and *RB1* in TP53^KO^ *vs*. TP53^WT^ oviduct-on-a-chip platforms. * indicates statistically significant changes (paired samples t-test, p < 0.05).

The *p53* relative expression was lower in the edited cells than in the control (paired samples t-test, p = 0.02). A decreased expression of *PTEN* and *RB1*, an increased expression of *Ki67*, *Myc*, and *PAX8* and no changes on *BRCA1/2* mRNAs were also observed (Fig. 4).

## Discussion

With approximately 239,000 new cases and 152,000 annual mortalities ovarian cancer is the most lethal gynecological cancer worldwide^52^. One of the main reasons for the slow progress in the understanding of the biology and evolution of epithelial ovarian cancers is that most tumor cells do not phenotypically resemble any type of cells from the ovary^3,6^. Ovarian HGSC is reported to originate from Fallopian tube secretory epithelial cells and the gene expression signature of HGSC is more closely related to Fallopian tube than to cells of the ovarian surface or endometrial surface^53^. Therefore, modeling transformation of Fallopian tube epithelium may be key to advancing our understanding of the genesis of ovarian cancer while confirming that STIC indeed progress to carcinoma. Here we used a large mammalian model (domestic dog) and developed a more user-friendly protocol to fabricate complex compartmentalized devices, such as the oviduct-on-a-chip, to successfully gene edit via CRISR-Cas9 and mimic STIC *in vitro*.

The development of compartmentalized microfluidic devices for organs-on-a-chip most often includes multiple rounds of design and fabrication, which is hampered by the technicality, cost and time duration of standard microfabrication strategies. Specifically, the incorporation of thermoplastic membranes between two PDMS layers has only been possible by using complex approaches, *e.g*., using spin-coated PDMS mortar^54^, SiO_2_ sputtering, silanization after plasma activation or corona treatment, and other chemical approaches^55^. In contrast, our partially curing approach only uses equipment ubiquitous to most laboratories and 3D printed molds, offering full versatility for changing designs, allowing the fabrication of compartmentalized devices in a short period of time (~24 h) and taking advantage of the growing online 3D printing market. Our microfluidics devices allowed dog oviductal epithelium growth, polarization and differentiation similarly to the *in vivo* oviduct epithelium. These results are in accordance with previous studies in porcine, murine, primates and bovine, in which the use of air-liquid interface (in Transwell) during the culture of oviductal epithelium promoted cell polarization and differentiation *in vitro*^56–58^.

Differently from these classical Transwell systems, the oviduct-on-a-chip allows the establishment of dynamic automated cultures with controlled flow and perfusion over the cellular models, which is of particular interest here for timely hormonal stimulation (mimicking the physiological pulsed release of hormones)^39^, which is not possible in a static Transwell system. Other advantages of organ-on-a-chip platforms include: the creation of a confined environment around the cell, as found *in vivo*; better control on any physical and chemical parameter in the cell vicinity, and this in a temporal manner; the possibility to generate physical and chemical gradients, which have been exploited for noninvasive studies of directional cell migration, differentiation, among other functions^39^. Furthermore, the reduced dimensions and volumes found in these organs-on-a-chip systems are advantageous since limited amounts of cells are required, which is beneficial when using rare cells, and for analysis of the cell activity since it is more concentrated than in conventional milliliter-sized systems. Finally, in our system it is possible to image cells *in situ* for tracking cell growth and migration, and our oviduct-on-a-chip platform could be coupled in the future to other organ-on-a-chip models of the female reproductive tract, such as the ovary, to investigate the interactions between these different organs.

Additionally, our highly biomimetic oviduct-on-a-chip permitted on-chip gene editing of *p53* by CRISPR-Cas9. CRISPR-Cas9 revolutionized the field of genome editing by specifically improving the efficiency of genetically engineered *in vitro* models. Delivering gRNA and purified Cas9 proteins complexes promotes lower off-target effect and higher cleavage efficiency than transfections using plasmid DNA^45^. Although electroporation can transfer gRNA-Cas9 complexes into cells with relatively high success, the use of lipid-mediated transfection is increasingly popular, due to its low cost, user-friendliness, and compatibility with high-throughput systems^45^. The delivery of protein complexes using lipid-mediated transfection was associated with high (up to 80%) modification efficiency in U2OS cell lines; however, it was hampered by significant cell toxicity when Lipofectamine 2000 was used^59^. Here we used Lipofectamine CRISPRMAX (Life Technologies), which has proven to be non-toxic to HEK293FT cells, mouse ES cells and human iPSCs, and to have a genome editing efficiency of 85, 75 and 55%, for these cell types, respectively^45^. Accordingly, the lipofection of Cas9 nuclease and gRNA complexes into our oviduct-on-a-chip yielded higher editing efficiency than when Fallopian tube epithelium was electroporated with Cas9-gRNA plasmids^7^.

The gene-edited dog oviduct-on-a-chip morphologically (loss of cell polarization and reduced ciliation, with increased cell atypia, proliferation, multilayered cell growth and DNA double-strand breaks)^2,13,46–51^ and genetically (increased *Ki67*, *PAX8* and *Myc* expression and decreased *PTEN* and *RB1* mRNA expression) mirrored the human STIC and HGSC. *Myc* oncogene amplification occurs in nearly half of HGSC tumors^60^, *RB1* down-regulation was detected in 67% of HGSC cases^61^, and *PAX8* was also up-regulated in HGSC^61^. *PTEN* loss was associated with hyperplasia and ovarian tumor formation^12^. *BRCA1* and *BRCA2* can sense DNA damage and are involved in DNA double-strand break (DBS) repair^62^. Moreover, STIC is normally accompanied by aggregation of the mutated p53 protein and an increased detection of *p53* expression in the oviductal epithelium^2,63,64^. However, similarly to our STIC-on-a-chip model, a reduction or absence of p53 protein is also observed in approximately 25% of STIC diagnosed patients^65^. Increases, reductions, or absence of *p53* expression are all considered surrogate markers for *TP53* mutations. Missense mutations are almost always associated with intense nuclear *p53* stain, whereas nonsense mutations encode truncated proteins that might not be recognized by the p53 antibody^65^ and, as such, could explain the reduced p53 protein detected in our TP53^KO^.

In summary, we proposed a rapid prototyping approach for the straightforward fabrication of compartmentalized microfluidic devices, in which cultured dog oviductal cells presented similar *in vivo* oviduct epithelium morphology and allowed on-chip gene editing by CRISPR-Cas9. In contrast to commonly studied rodents, the domestic dog is a better model because dogs (1) spontaneously develop ovarian cancer and (2) share similar disease hallmarks, expression and mutation of key genes with humans^20,21,25,27,66–69^. The gene-edited dog oviduct-on-a-chip will open new opportunities for understanding STIC progression to HGSC, to the identification of biomarkers for the early diagnosis of this lethal disease and will be instrumental in developing patient-derived *in vitro* STIC models, which is essential to support the paradigm of personalized medicine.

## Materials and Methods

### Chip design and fabrication

Devices were designed using SolidWorks: the top compartment was a double elongated channel (3 mm wide, 24 mm long and 4 mm high), the bottom compartment being identical but with a smaller height (1 mm). Molds for soft-lithography were printed using an SLA FlashForge Hunter printer (FlashForge, Jinhua, China) using Fun-To-Do Industrial Blend resin (Fun-To-Do, Alkmaar, The Netherlands), and post-cured under a 405 nm UV light at 14 mW cm^−2^ for 2 h followed by 24 h at 60 °C. PDMS with a pre-polymer:curing agent 15:1 (Sylgard 184, Dow Corning Midland, United States) were poured on the 3D printed molds and partially cured for 25 min at 62 °C in an oven. The top and bottom compartments were peeled off the mold, and horizontal inlets and outlets were created in both the top and bottom parts using a 1.5-mm biopsy punch (Integra® Miltex®, USA). A porous polycarbonate membrane (Nuclepore™ Track-Etched Membranes, pore size: 0.4 µm, Whatman®, USA) was sandwiched between the aligned top and bottom parts and devices were fully cured at 62 °C in an oven overnight. After curing, silicone tubes (o.d. 1.5 mm, i.d. 0.5 mm, Tygon tubing, Cole-Parmer®, USA) were secured in the inlets and outlets using PDMS glue (10:1 pre-polymer:curing agent ratio), which was cured for 30 min at 62 °C.

### Oviduct collection and culture in the microfluidic devices

All female reproductive tracts were opportunistically collected from local veterinary clinics after routine spaying procedures of household and stray dogs. No additional permissions were required since these biological materials were designated for disposal via incineration. Oviducts of adult domestic dogs (>1 year old, n = 16) were recovered and transported (at 4 °C) to the laboratory within 6 h of excision. After being isolated from surrounding tissues, a 23 G needle was inserted through the fimbria opening and the entire oviduct was infused with TripLE Express (~100 µL; Thermo Scientific, USA) and kept at 38.5 °C, 5% CO_2_ in a humidified incubator, immersed in DMEM/Ham’s F12 medium (DMEM/F12 Glutamax I, Gibco, USA) supplemented with 100 µg ml^−1^ gentamycin (Thermo Scientific), 5% fetal calf serum (FCS, Sigma-Aldrich, USA) and 2.5 mg ml^−1^ amphotericin B (Thermo Scientific) - flush medium, for 1 h. The oviducts were then flushed with 3 mL of flush medium and centrifuged at 3,000 rpm for 5 min, when supernatant was removed and cells resuspended in culture medium (flush medium with 10% FCS). Total isolated cells were seeded in a 6-well plate (Costar Corning Incorporated, USA) containing 4 mL of culture medium and cultured overnight at 38.5 °C, 5% CO_2_ in a humidified incubator. During this period, the cells formed floating vesicles with outward beating cilia (Supplementary Video S1). These vesicles were collected, centrifuged at 3,000 rpm for 10 min, suspended in Opti-MEM™ I Reduced Serum Media (GIBCO) with 100 µg ml^−1^ gentamycin and 10% FCS (CRISPR culture medium), and pipetted up and down several times to mechanically separate the cells. Next, cells from four different donor animals were mixed and seeded into the microfluidic devices at a concentration of 10^6^ cells ml^−1^ and allowed to attach and reach 50–70% confluence during 3–4 days at 38.5 °C, 5% CO_2_ in a humidified incubator. At day 4 perfusion started in the bottom compartment of the oviduct-on-a-chip at a flow rate of 5 µl h^−1^ ^37^. In a preliminary study, cells were cultured for up to 3 weeks and analyzed for morphology as described below. Pooled freshly isolated cells were also frozen at −20 °C for mRNA analysis.

### Synthetic guide RNA design and construction

Two synthetic guide RNAs (sgRNA) were designed using Synthego CRISPR Design tool for the tumor protein *p53* gene (TP53, Gene ID: 403869) of the dog (*Canis lupus familiaris*), in exon 4: (1) G*A*C*CGUCCAAGUAACAGACU and (2) A*G*C*CAAGUCUGUUACUUGGA. Both sgRNAs were synthesized by Synthego (USA).

### Gene editing of the oviduct-on-a-chip by lipofection

After reaching 50–70% confluence in the microfluidic device, cells were washed with FCS-free and antibiotics-free CRISPR culture medium, and 200 µl of same medium was added to the top compartment of the oviduct-on-a-chip. Immediately before transfection, 300 ng of Cas9 protein (Gene Art Platinum Cas9 nuclease, Thermo Fisher Scientific) and 75 ng of each sgRNAs (1 and 2) were mixed in 50 µl of Opti-MEM medium, 1 µl of Cas9 Plus reagent (Thermo Fisher Scientific) was added, briefly vortexed and incubated for 5 min at room temperature to allow the formation of Cas9 and gRNAs complexes (Cas9 RNPs). Meanwhile, lipofectamine solution was prepared by adding 3 µl of Lipofectamine CRISPRMAX (Thermo Fisher Scientific) reagent to 50 µl of Opti-MEM, briefly vortexing and incubating for 5 min at room temperature. The Cas9 RNPs were finally added (dropwise) to the Lipofectamine CRISPRMAX solution, the mixture was incubated for 15 min at room temperature and added to the cells. As negative control, cells were incubated with the same transfection solution without sgRNAs. At 72 h post-transfection cells were washed with Phosphate Buffer Saline (PBS, GIBCO) and 1) either they were lysed with 200 µl of buffer RLT plus (QIAGEN, USA) to be used for DNA, RNA and protein extraction, or 2) medium was removed from the top (air interface), cultured for 7 days under perfusion of the basolateral compartment (5 µl h^−1^) and fixed in 4% paraformaldehyde for immunofluorescence.

### Immunofluorescence of the oviduct-on-a-chip

After fixation, the cells were permeabilized for 30 min using 0.5% Triton-X100 in PBS. Non-specific binding was blocked by incubation for 1 h in PBS containing 5% normal goat serum (Sigma-Aldrich) at room temperature. The cells were then incubated for 1 h at room temperature with a monoclonal mouse anti-acetylated α-tubulin (1:100, Santa Cruz Biotechnology, USA) for morphology; or with a monoclonal mouse anti-Ki67 antigen clone MIB-1 (ready to use, Dako Omnis, USA) for cell proliferation; or with a monoclonal mouse anti-p-Histone H2A.X (yH2AX, 1:100, Santa Cruz Biotechnology) for double strand breaks. Next, the cells were washed and incubated with an Alexa 488 conjugated goat anti-mouse antibody (1:200, Molecular Probes, USA) for 1 h. Hoechst 33342 (5 µg ml^−1^, Molecular Probes) was used to stain cell nuclei and phalloidin conjugated to Alexa 568 (1:100, Molecular probes) was used to stain actin filaments. Cells were visualized by a fluorescence microscope (EVOS FL auto 2, Invitrogen, USA). To evaluate Ki67 and yH2AX expression, a total of ten randomly selected areas were imaged per device and at least 300 cells were counted. ﻿Moreover, Z-stacks of 0.1 µm were obtained using a 100× objective. Side-views of the cells were re-constructed using ImageJ software (National Institutes of Health, Bethesda, MD, USA) to show cell morphology and at least 100 cells per device were measured to determine population cell height.

### DNA, RNA and protein extraction

After lysis, the cell suspension was vortexed for 1 min, placed in a DNA spin column (DNeasy blood and tissue kit, QIAGEN) and centrifuged for 30 s at 10,000 rpm. DNA spin column was used for genomic DNA collection as described by the manufacturer; 200 µl of 70% ethanol was added to the flow, mixed by pipetting and transferred to a RNeasy MinElute spin column (RNeasy Plus Micro kit, QIAGEN) placed in a 2 ml collection tube and centrifuged at 10,000 rpm for 15 s. RNA column was used for RNA isolation as described by manufacturer and flow was added to 4.8 ml of ice cold acetone, incubated on ice for 30 min, centrifuged at 10,000 rpm for 10 min to pellet the proteins. Total pelleted proteins were resuspended in 20 µl of RIPA lysis buffer (Santa Cruz Biotechnology). Eluted RNA, DNA and proteins were stored at −20 °C for a short period (<1 week) until use.

### PCR and Sanger sequencing of gDNA

Genomic DNA (gDNA, 10 ng) was amplified by PCR using the AmpliTaq Gold 360 Master Mix (Applied Biosytems) according to manufacturer’s protocol, and using 100 µM of both forward and reverse primers for the *p53* gene (Table 1). PCR primers for Sanger sequencing were designed to include at least 100 bases before and after the region of the sgRNAs cut. The DNA sequences of the amplified region were verified using the Sanger sequencing conducted at the Bioanalytical services lab of the Institute of Marine and Environmental Technology, University of Maryland Center for Environmental Science.

**Table 1:** Primers used in the study for Sanger sequencing and RT-PCR.

Gene name	Gene ID*	Primer sequence	
Tumor protein *p53* (TP53)	403869	*F*	5′*–*CCAGAGAGCGTCGTGAACTG–3′
		R	5′*–*CTTGGCCAAATTTCCTT–3′
Myc proto-oncogene (*Myc*)	403924	*F*	5′*–AAAAGGTCCGAATCGGGGTC*–3′
		R	5′*–*ATCTGATCACGCAGGGCAAA–3′
Phosphatase and tensin homolog (*PTEN*)	403832	*F*	5′*–CCAATTCAGGACCCACACGA*–3′
		R	5′*–*CTAGCCTCCGGGTTTGATGG–3′
RB transcriptional corepressor 1 (*RB1*)	476915	*F*	5′*–AGGCAGCAACCCTCCTAAAC*–3′
		R	5′*–*TCCGGGACCCTCATTTCTCT–3′
Paired box 8 (*PAX8*)	403927	*F*	5′*–ACAAACGGCAGAACCCTACC*–3′
		R	5′*–*GTCGTTGTCACAGACACCCT–3′
Marker of proliferation KI-67 (*Ki67*)	100686578	*F*	5′*–GGTCGTCTGAAACCGGAGTT*–3′
		R	5′*–*GAGCTGGAGATCCCTTACGC–3′
*GAPDH*	403755	*F*	5′*–GTAGTGAAGCAGGCATCGGA*–3′
		R	5′–GTCGAAGGTGGAAGAGTGGG–3′
*BRCA1*	403437	*F*	5′*–AGGAAAGCGCGGGAATTACA*–3′
		R	5′–GCCCTCACTCAAGAAAGCCT–3′
*BRCA2*	474180	*F*	5′*–GTCAGCTTTCTGGCCGAAGT*–3′
		R	5′*–*AATCTGCTTGATTGCACCGC–3′

*from Canis lupus familiaris.

### Reverse transcription and RT-PCR

A total of 30 ng of extracted mRNA were reversible transcribed using the Superscript III First-Strand Synthesis SuperMix (Thermo Fisher), following manufacturer’s instructions. Primers for Phosphatase and tensin homolog (*PTEN*), Myc proto-oncogene (*Myc*), RB transcriptional corepressor 1 (*RB1*), Paired box 8 (*PAX8*), Marker of proliferation Ki-67 (*Ki67*), *BRCA1* and *BRCA2* were designed using Prime-BLAST (Primer3 and BLAST, NCBI, USA) and the *Canis lupus familiaris RefSeq* mRNA (Table 1). mRNA expression was quantified through RT-PCR using a 1 µL of total cDNA and SYBR Green PCR Master Mix (Applied Biosystems, Life Technologies, USA), following manufacturer’s instructions. The program started with 10 min at 95 °C followed by 40 cycles each of 10 s at 95 °C and 60 s at 60 °C. Melting curves were plotted after each cycle series. A standard curve of cDNA, with a 3-fold dilution series was obtained by plotting the log of the starting amount against the cycle threshold value of the dilution series. GAPDH was used as a reference gene for normalization of expression levels^70^.

### Western blot

Whole-cell lysates were collected and processed as described above, and total collected proteins were boiled at 95 °C for 5 min and separated by electrophoresis using a 4–20% Mini-Protean TGX Precast gel (Bio-Rad, USA). Immediately after electrophoresis proteins were transferred to a Nitrocellulose membrane (Trans-Blot Turbo Mini Nitrocellulose transfer packs, Bio-Rad). After blotting, the membranes were washed in blocking solution (Thermo Scientific Pierce, Clear Milk Blocking Buffer) for 1 h at room temperature. Membrane was then incubated overnight at 4 °C with mouse monoclonal anti-p53 antibody (Pab 240, Abcam, USA), at a dilution of 1:1,000. Next, membranes were washed and incubated for 1 h with goat anti-mouse IgG (H + L)-HRP conjugate (Bio-Rad) diluted at 1:10,000; and proteins were detected using the SuperSignal West Pico Plus chemiluminescent substrate (Thermo Fisher) in a G:Box (Syngene). After imaging p53 bands, the membranes were stripped using the Restore Plus Western Blot Stripping Buffer (Thermo Fisher), and loading was confirmed by repeating the western blot process using a polyclonal rabbit anti-beta actin primary antibody (1:1,000, Cell Signaling Technology, USA) and a goat anti-rabbit IgG (H + L)-HRP conjugate (Bio-Rad) secondary antibody diluted at 1:10,000. Lanes ran without protein, as well as protocol performed in the absence of primary antibody were used as controls for p53 antibody specificity, and no bands were observed.

### Statistical analysis

The data were analyzed using IBM SPSS Statistics (version 24) and GraphPad (version 6, Prism). Data are presented as mean ± standard deviation. A paired samples t-test with 95% confidence interval was used to compare protein expression of TP53, Ki-67 and yH2AX expression, the mRNA expression and the number of giant multi-nucleated cells between groups.

## Supplementary information

Supplementary video S1.

Supplementary files.

## Data Availability

The authors declare that all available data are present in the manuscript and Supplementary Files.
