# Author Correction: A dog oviduct-on-a-chip model of serous tubal intraepithelial carcinoma

**DOI:** 10.1038/s41598-020-61782-w

**Published:** 2020-03-10

**Authors:** Marcia de Almeida Monteiro Melo Ferraz, Jennifer Beth Nagashima, Bastien Venzac, Séverine Le Gac, Nucharin Songsasen

**Affiliations:** 1 Center for Species Survival, Smithsonian National Zoo and Conservation Biology Institute, 1500 Remount Road, Front Royal, Virginia 22630 USA; 2 Applied Microfluidics for Bioengineering Research, MESA+ Institute for Nanotechnology and TechMed Center, University of Twente, 7500 AE Enschede, The Netherlands

Correction to: *Scientific Reports* 10.1038/s41598-020-58507-4, published online 31 January 2020

This Article contains an error in Figure 4 where the labels PAX8 and PTEN on the x-axis were reversed. The correct Figure 4 appears below as Figure 1.

**Figure 1:**
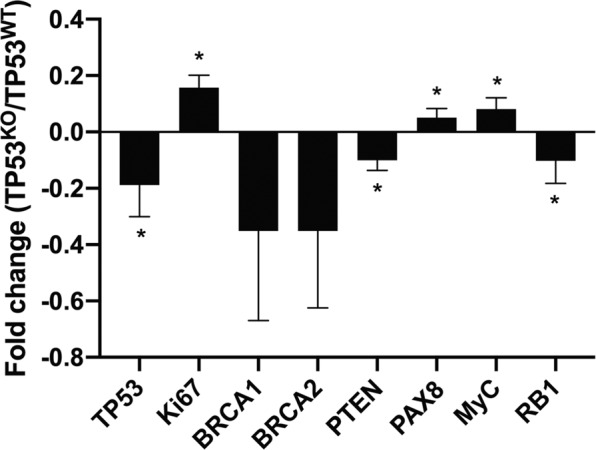
.

