# Supplementary material for: A dog oviduct-on-a-chip model of serous tubal intraepithelial carcinoma
Source: Sci Rep. 2020 Jan 31;10:1575. doi: 10.1038/s41598-020-58507-4 (PMC6994655; doi:10.1038/s41598-020-58507-4)
Supplement: Supplementary file 2 — Supplementary files. [file 41598_2020_58507_MOESM2_ESM.docx]

**A dog oviduct-on-a-chip model of serous tubal intraepithelial carcinoma**

Marcia de Almeida Monteiro Melo Ferraz*^a^, Jennifer Beth Nagashima^a^, Bastien Venzac^b^, Séverine Le Gac^b^ and Nucharin Songsasen^a^

^a^Center for Species Survival, Smithsonian National Zoo and Conservation Biology Institute, 1500 Remount Road, Front Royal, Virginia 22630, USA.

^b^Applied Microfluidics for Bioengineering Research, MESA+ Institute for Nanotechnology and TechMed Center, University of Twente, 7500 AE Enschede, The Netherlands

## *Corresponding author

[ferrazm@si.edu](mailto:ferrazm@si.edu)

+1 (540) 635-0494

**Supplementary files**

**Supplementary Table S1**. Top 10 off-target sites of sgRNA 1 and 2 as determined by BLAST alignment.

| **sgRNA** | **BLAST score** | **SEQUENCE** | **Mismatches** | **GENE** |
| --- | --- | --- | --- | --- |
| **1**  **2** | 28.2  30.2 | **CCAAGTAACAGACT**  **AAGTCTGTTACTTGG** | 3  2 | GRM7 |
| **1**  **2** | 26.3  26.3 | **CCAAGTAACAGAC**  **GTCTGTTACTTGG** | 4  4 | METTL21A |
| **1** | 26.3 | **GTCCAAGTAACAG** | 4 | [EOGT](https://design.synthego.com/#/design/results?genome=canis_familiaris_e87&nuclease=cas9&symbol=PXDNL) |
| **1** | 26.3 | **GTCCAAGTAACAG** | 4 | [ANKRD6](https://design.synthego.com/#/design/results?genome=canis_familiaris_e87&nuclease=cas9&symbol=WHSC1) |
| **1** | 26.3 | **CGTCCAAGTAACA** | 4 | [TAOK1](https://design.synthego.com/#/design/results?genome=canis_familiaris_e87&nuclease=cas9&symbol=unknown) |
| **1**  **2** | 26.3  26.3 | **CAAGTAACAGACT**  **AGTCTGTTACTTG** | 4  4 | [HECTD1](https://design.synthego.com/#/design/results?genome=canis_familiaris_e87&nuclease=cas9&symbol=PARK7) |
| **1**  **2** | 26.3  26.3 | **CCAAGTAACAGAC**  **GTCTGTTACTTGG** | 4  4 | ZNF446 |
| **1**  **2** | 26.3  26.3 | **CAAGTAACAGACT**  **AGTCTGTTACTTG** | 4  4 | [URI1](https://design.synthego.com/#/design/results?genome=canis_familiaris_e87&nuclease=cas9&symbol=KDM4B) |
| **1**  **2** | 26.3  30.2 | **CAAGTAACAGACT**  **CAAGTCTGTTACTTG** | 4  2 | ZNF154 |
| **2** | 26.3 | **CAAGTCTGTTACT** | 4 | FSD2 |

**Supplementary Table S2**. Off-target sites of sgRNA 1 as determined by the CRISPR design tool (Synthego, USA)

| **OFF-TARGET SITE** | **MISMATCHES** | **CHROMOSOME** | **CUT SITE** | **PAM** | **GENE** |
| --- | --- | --- | --- | --- | --- |
| **AGCCAACTCTGCTACTTGCA** | 3 | 14 | 46,001,835 | GGG | [BMPER](https://design.synthego.com/#/design/results?genome=canis_familiaris_e87&nuclease=cas9&symbol=BMPER) |
| **AGCCAAGTCTGTAACAGGGA** | 3 | 25 | 43,158,626 | GGG | [ARMC9](https://design.synthego.com/#/design/results?genome=canis_familiaris_e87&nuclease=cas9&symbol=ARMC9) |
| **ATCCAAGTCTGTTATTTGGT** | 3 | 29 | 3,544,898 | AGG | [PXDNL](https://design.synthego.com/#/design/results?genome=canis_familiaris_e87&nuclease=cas9&symbol=PXDNL) |
| **AGCCAAGTCGGGGACTTGGA** | 3 | 3 | 62,174,179 | GGG | [WHSC1](https://design.synthego.com/#/design/results?genome=canis_familiaris_e87&nuclease=cas9&symbol=WHSC1) |
| **ATCCAAGTCTTTAACTTGGA** | 3 | 18 | 35,492,397 | GGG | [unknown](https://design.synthego.com/#/design/results?genome=canis_familiaris_e87&nuclease=cas9&symbol=unknown) |
| **AGCAAGGTCTGTTACTTAGA** | 3 | 5 | 61,586,947 | AGG | [PARK7](https://design.synthego.com/#/design/results?genome=canis_familiaris_e87&nuclease=cas9&symbol=PARK7) |
| **AGCCAGGTCTGCTCCTTGGA** | 3 | X | 32,507,539 | TGG |  |
| **AGACAAGTCTCTTACTTGGT** | 3 | 20 | 54,792,816 | AGG | [KDM4B](https://design.synthego.com/#/design/results?genome=canis_familiaris_e87&nuclease=cas9&symbol=KDM4B) |
| **TGCCAAGTCAGTTACATGGA** | 3 | 19 | 9,227,976 | AGG |  |
| **TGCCAAGTCAGTTACATGGA** | 3 | 19 | 9,228,054 | AGG |  |
| **AGTCAAGTCTGTGACCTGGA** | 3 | 19 | 46,250,836 | AGG | [GTDC1](https://design.synthego.com/#/design/results?genome=canis_familiaris_e87&nuclease=cas9&symbol=GTDC1) |
| **TGCCAAGTCTGGAACTTGGG** | 4 | 14 | 46,943,705 | AGG | [TBX20](https://design.synthego.com/#/design/results?genome=canis_familiaris_e87&nuclease=cas9&symbol=TBX20) |
| **AGCAATGTCTGTTACGTGGG** | 4 | 14 | 47,278,880 | TGG | [HERPUD2](https://design.synthego.com/#/design/results?genome=canis_familiaris_e87&nuclease=cas9&symbol=HERPUD2) |
| **AGCAAATTCTGTTACTTCGT** | 4 | 14 | 48,279,157 | TGG | [ELMO1](https://design.synthego.com/#/design/results?genome=canis_familiaris_e87&nuclease=cas9&symbol=ELMO1) |
| **AGCCAAGACTGTTGCTGAGA** | 4 | 7 | 49,343,358 | AGG |  |
| **ACTCCTGTCTGTTACTTGGA** | 4 | 7 | 62,816,283 | TGG |  |
| **AGCCAAGTCTGTAAGAGGGA** | 4 | 37 | 9,266,097 | AGG |  |
| **AGCTGAGTCAGTAACTTGGA** | 4 | 37 | 15,821,218 | TGG |  |
| **GGCAAAGTCTGTTGCTTAGA** | 4 | 24 | 14,000,178 | GGG |  |

**TOTAL OFF-TARGETS FOUND: 0,0,0,11,114**

**Supplementary Table S3**. Off-target sites of sgRNA 2 as determined by the CRISPR design tool (Synthego, USA)

| **OFF-TARGET SITE** | **MISMATCHES** | **CHROMOSOME** | **CUT SITE** | **PAM** | **GENE** |
| --- | --- | --- | --- | --- | --- |
| **GAACGTACTAGTAACAGACT** | 3 | 4 | 34,730,992 | GGG | [FAM35A](https://design.synthego.com/#/design/results?genome=canis_familiaris_e87&nuclease=cas9&symbol=FAM35A) |
| **GACCCTTCAAGTCAGAGACT** | 4 | 16 | 47,822,827 | TGG | [TENM3](https://design.synthego.com/#/design/results?genome=canis_familiaris_e87&nuclease=cas9&symbol=TENM3) |
| **GGCCGACCAAGTAAAAGACA** | 4 | 6 | 22,549,188 | TGG |  |
| **AGCTGTCCAAGTCACAGACT** | 4 | 6 | 29,641,090 | GGG |  |
| **GCGCGTCCAAGTATCACACT** | 4 | 14 | 56,692,226 | TGG |  |
| **GACCGTGGGAGGAACAGACT** | 4 | 14 | 58,896,277 | TGG |  |
| **GACAAACCAAGAAACAGACT** | 4 | 16 | 21,725,249 | CGG | [unknown](https://design.synthego.com/#/design/results?genome=canis_familiaris_e87&nuclease=cas9&symbol=unknown) |
| **GACCTTCCAAGAAATGGACT** | 4 | 35 | 15,934,345 | TGG | [unknown](https://design.synthego.com/#/design/results?genome=canis_familiaris_e87&nuclease=cas9&symbol=unknown) |
| **AACCGTGCAAGGAAAAGACT** | 4 | 35 | 22,247,572 | TGG |  |
| **CTCCGTCCAAGTATCAGAGT** | 4 | 10 | 45,787,157 | CGG | [THADA](https://design.synthego.com/#/design/results?genome=canis_familiaris_e87&nuclease=cas9&symbol=THADA) |
| **CACTGTCCAAGTTACAGATT** | 4 | 20 | 44,985,288 | AGG | [unknown](https://design.synthego.com/#/design/results?genome=canis_familiaris_e87&nuclease=cas9&symbol=unknown) |
| **GACTGTCCCAGGCACAGACT** | 4 | 14 | 47,721,538 | AGG | [EEPD1](https://design.synthego.com/#/design/results?genome=canis_familiaris_e87&nuclease=cas9&symbol=EEPD1) |
| **GACCAGCCAAGTAACAGAAG** | 4 | 11 | 49,569,140 | TGG |  |
| **GACAAACCAAGAAACAGACT** | 4 | 1 | 47,005,592 | TGG | [ZDHHC14](https://design.synthego.com/#/design/results?genome=canis_familiaris_e87&nuclease=cas9&symbol=ZDHHC14) |
| **GTCCTTGCAAGTAACAGTCT** | 4 | 1 | 58,446,110 | AGG |  |
| **GACCCACGAAGTCACAGACT** | 4 | 2 | 23,103,250 | TGG |  |
| **CACTGCCAAAGTAACAGACT** | 4 | 2 | 35,552,026 | AGG | [HBEGF](https://design.synthego.com/#/design/results?genome=canis_familiaris_e87&nuclease=cas9&symbol=HBEGF) |
| **GACAGACCAAGTAATAAACT** | 4 | 5 | 44,816,036 | TGG |  |
| **GGCCCTCCAGGTAACCGACT** | 4 | 13 | 45,605,898 | GGG | [SCFD2](https://design.synthego.com/#/design/results?genome=canis_familiaris_e87&nuclease=cas9&symbol=SCFD2) |

##### TOTAL OFF-TARGETS FOUND: 0,0,0,1,29


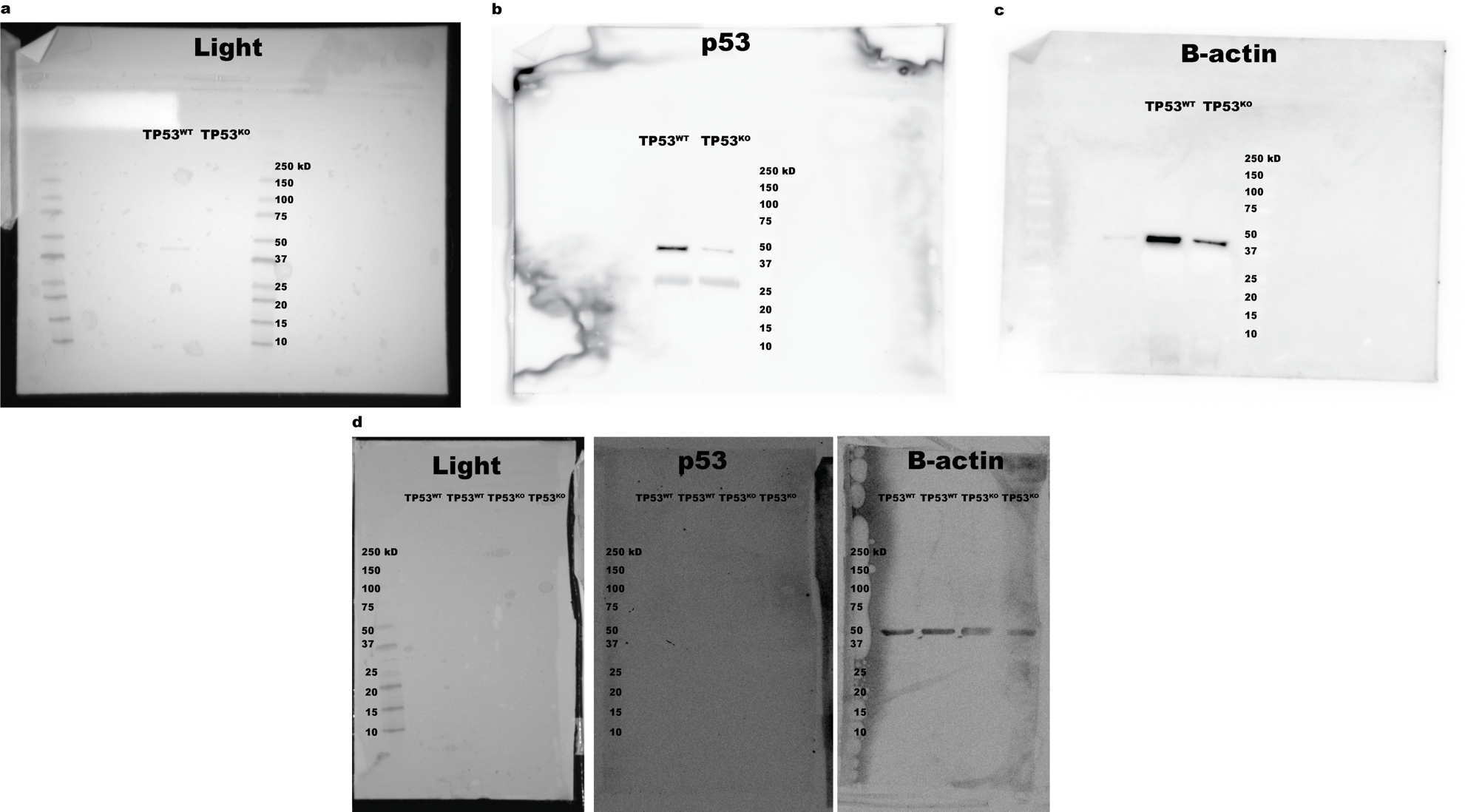


**Supp. Fig. S1**. Full image of Western blot membranes. In **a**, light image of membrane showing the protein standard (Precision Plus Protein™ Dual Color Standards, Bio-Rad). In **b**, p53 staining, showing a main band around 50kDa. In **c**, B-actin staining as loading control, showing single bands around 42kDa. And **d**, negative control (omitting p53 primary antibody), to check specificity of secondary antibody (middle image) and B-actin staining as loading control of lanes (right image).


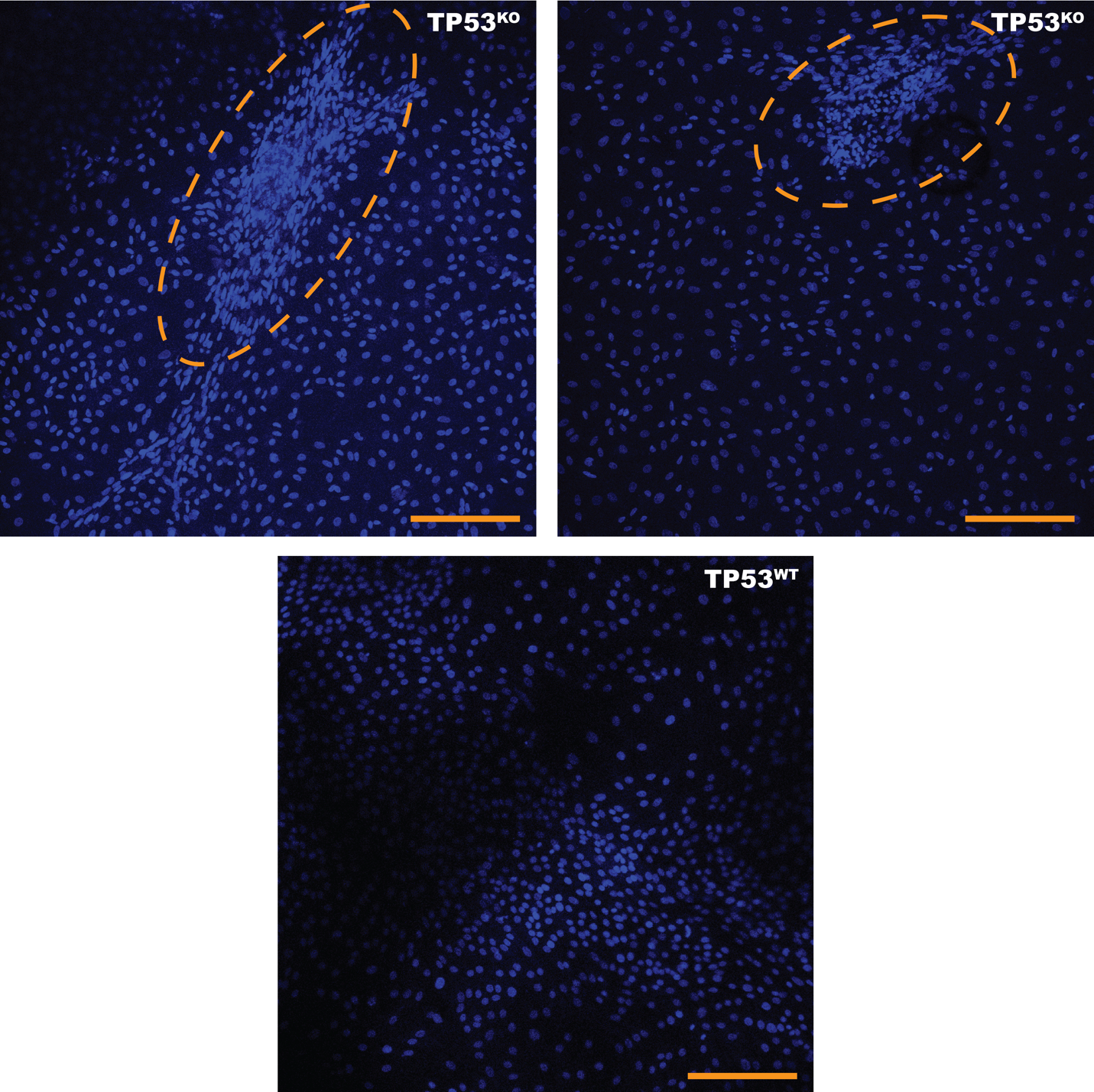


**Supp. Fig. S2**. On chip imaging of TP53^KO^ (**a** and **b**) and TP53^WT^ oviductal cells at day 14 of cell culture, non-fixed cells were stained with Hoechst 33342 (5 µg ml^−1^) for 15 min previous to imaging. Note areas of multicellular layer growth in TP53^KO^ cultures. Bars = 100 µm

**Supp. Video S1**. Live video of dog oviductal epithelial cells after initial 24 h of culture.
